# Evaluation of Mass Drug Administration Coverage for Lymphatic Filariasis in the Lukonga Health Zone in 2022

**DOI:** 10.3390/tropicalmed9070156

**Published:** 2024-07-11

**Authors:** Patrick N. Ntumba, Pierre Z. Akilimali

**Affiliations:** 1 Kinshasa School of Public Health, University of Kinshasa, Kinshasa P.O. Box 11850, Democratic Republic of the Congo; ntumbap980@gmail.com; 2 Department of Nutrition, Kinshasa School of Public Health, University of Kinshasa, Kinshasa P.O. Box 11850, Democratic Republic of the Congo; 3 Patrick Kayembe Research Center, Kinshasa School of Public Health, University of Kinshasa, Kinshasa P.O. Box 11850, Democratic Republic of the Congo

**Keywords:** lymphatic filariasis, therapeutic coverage, supervised treatment, mass treatment, preventive chemotherapy

## Abstract

(1) Background and rationale: To validate the reported therapeutic coverage, a lymphatic filariasis post-mass drug administration (MDA) campaign survey was conducted in the Lukonga health zone from 10 June to 15 July 2023. (2) Materials and methods: This was a descriptive, cross-sectional study conducted at the community level in 30 villages in the Lukonga health zone from 10 June to 15 July 2023. The study population included all individuals from the visited communities. The study variables included age, sex, drug use (ivermectin + albendazole), adverse events, and adherence to MDA guidelines for supervised drug use. Questionnaires were administered on Android phones using the SurveyCTO platform. Stata version 17 was used for data analysis. (3) Results: Of the 1092 respondents, 54.8% were female and one-third were between the ages of 5 and 14. Two-thirds of the households surveyed, or 64%, had more than six people living in them, and 1031 individuals, or 94%, reported being present during the community mass drug distribution. Notably, 678 individuals, or 66%, reported taking the drugs offered, and 66.4% of those who took the drugs reported doing so in the presence of drug distributors. Thus, the survey coverage was 65.7% [95% CI: 62.9–68.7]. The results of this study show that the survey coverage was above the 65% threshold recommended by the WHO but below the 82.3% reported by the Lukonga health zone. The main reason for non-compliance was a fear of ivermectin-related side effects (47%). Supervised or directly observed treatment was not adhered to (66.4%). (4) Discussion and conclusions: Key challenges to further increase treatment coverage include assessing data quality, building capacity, motivating drug distributors, improving data reporting tools, proper recording by drug distributors, and accurate reporting on non-residents who take the drugs during the MDA. In addition, harmonization of the numerator for calculating drug coverage in the health zone is critical. It is imperative to provide the public with explicit information regarding the objective of drug distribution and the probable adverse effects.

## 1. Background

Lymphatic filariasis (LF) is a preventable, disabling, and disfiguring disease caused by infection with parasitic filarial worms of the species *Wuchereria bancrofti* (Cobbold, 1877), *Brugia malayi* (Brug, 1927), and *B. timori* Partono, Purnomo, Dennis, Atmosoedjono, Oemijati and Cross, 1977. It is estimated that 51.4 million people worldwide were infected in 2020 [1]. Lymphedema and hydrocele are the visible chronic clinical consequences of lymphatic damage caused by the presence of these parasites in the body. The parasites are transmitted from person to person by mosquitoes of the genera Culex, Anopheles, Mansonia, and Aedes [1]. Lymphatic filariasis can be eliminated by stopping the spread of the infection through chemoprevention. Mass drug administration (MDA) is a WHO-recommended chemoprevention strategy for the elimination of lymphatic filariasis. It involves administering an annual dose of drugs to the entire at-risk population. The drugs used have a limited effect on adult parasites, but they effectively reduce the density of microfilariae in the blood, thus preventing the transmission of parasites to mosquitoes [2]. Since the year 2000, a total of over 8.6 billion treatments have been administered through mass drug administration (MDA) to a population exceeding 925 million individuals [1]. In 2020, the global population in need of mass drug administration (MDA) was 863.2 million [1]. Out of this, 28 countries reported successfully treating 358.8 million individuals, which accounted for 41.6% of the total population requiring MDA [1]. Recent research suggests that lymphatic filariasis transmission in at-risk populations has decreased by 43% since the global program began [3]. In 2018, it was estimated that 49 countries would need MDA. To achieve the elimination targets, MDA must be conducted consecutively each year in each endemic implementation unit (IU), with at least one effective coverage of the entire population [4].

To interrupt the transmission of LF in endemic countries, it is recommended that effective antifilarial drugs be administered en masse to the entire population at risk for a sufficient duration. The effectiveness of MDA in reducing the prevalence and density of microfilariae is directly related to the percentage of the population that takes the drugs each year. It is estimated that the total population coverage must be at least 65% to be effective [5]. This is the minimum process indicator for evaluating the results of large-scale chemoprevention interventions. It is recognized that post-MDA coverage evaluations are an important component of NTD programs. Coverage assessment surveys are a valuable tool for evaluating program performance. These surveys are population-based and designed to provide accurate estimates of PC coverage while overcoming many of the biases and errors that can affect reported coverage. Although generally considered a tool for estimating PC coverage, the benefits and uses of coverage evaluation surveys extend beyond estimating treatment coverage [6].

In theory, coverage refers to the proportion of people in the population or target group who have actually taken the recommended drug or combination of drugs. While reported coverage is an essential tool for program monitoring, it is subject to error due to inaccurate estimates of the target population, weak health information systems, underreporting, and intentional inflation of the number of people treated [7,8]. For this reason, the Global Program to Eliminate LF recommends that a population-based coverage survey be conducted regularly after each MDA round to validate the reported coverage and better assess the program’s performance. Treatment coverage from population surveys provides a more reliable estimate and is not affected by missing data, compilation errors, or difficulties in estimating an accurate denominator from the census data [9]. The results of the coordinated and integrated mapping conducted by the National Coordination of Tropical Diseases from 2009 to 2015 revealed that lymphatic filariasis is prevalent in the Democratic Republic of Congo (DRC), with an estimated 39.7% of the population at risk of developing the infection as measured by microfilaraemia [10]. The Lukonga health zone in the Kasaï Central province is not exempt from this problem; it is endemic for lymphatic filariasis, with infection prevalence rates of 0.03 per thousand inhabitants in 2020 and 2021, and 0.23 per thousand inhabitants in 2022. In 2022, 82.3% of the population was reached during the mass drug administration campaign.

The DRC, like other member countries of the World Health Organization (WHO) African Region (WHO-AFRO), has committed to combating these diseases through the National Program for the Control of Neglected Tropical Diseases with Preventive Chemotherapy (PNLMTN-CTP). Consequently, all populations residing in endemic areas have been treated through mass drug administration (MDA) campaigns on an annual basis over the past decade [10]. To guarantee the monitoring and evaluation of interventions, it is recommended that coverage evaluations of mass distributions be conducted. These evaluations will validate the reported coverages and allow for refinement of the mass drug distribution strategies. The objective of this study was to evaluate the extent of coverage, adherence, and reasons for lack of adherence to MDA in the Lukonga district of Kasaï Central, which is known for high prevalence of the disease.

## 2. Materials and Methods

### 2.1. Study Framework

**Figure 1:**
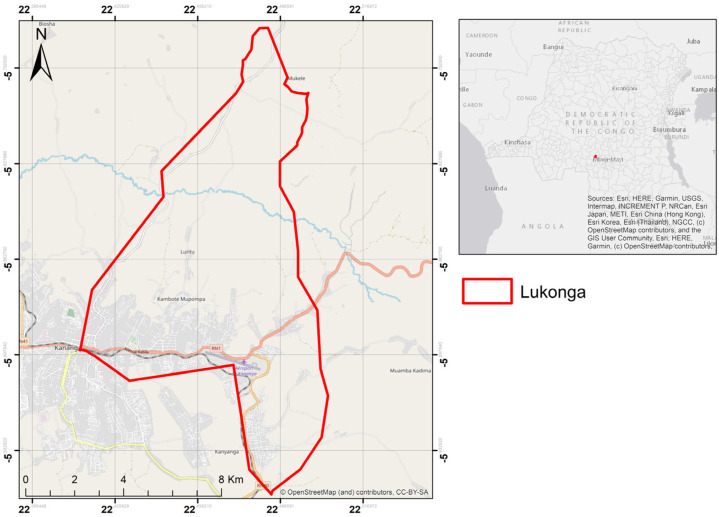
Lukonga Health Zone.

This study was conducted in the urban–rural health zone of Lukonga (See Figure 1), located in the city of Kananga and part of the 26 health zones within the Kasaï Central Provincial Health Division (DPS). The health zone covers an area of 172 km^2^ and has a population of 348,926 inhabitants. The Lukonga health zone is bordered to the north by the Demba health zone, to the south by the Ndesha health zone, and to the east by the Kananga health zone. The population of the Lukonga health zone has the lowest socio-economic status in the city, with an average daily income of less than USD 1. This is reflected in the low purchasing power. Lukonga has 19 health areas.

### 2.2. Study Design

A descriptive cross-sectional survey was conducted in 30 villages of the Lukonga health zone from 10 June 2023 to 15 July 2023. The 30 villages chosen are situated in 10 out of the 19 health areas. This was five months after the mass treatment in accordance with the WHO guidelines recommending that coverage surveys be conducted within six months following the distribution to avoid recall bias [6].

### 2.3. Study Population

The study population included individuals, regardless of their eligibility for MDA against LF, who were present or absent on the day of the survey and resided in households during the mass treatment period (December 2022).

### 2.4. Sampling

The Coverage Survey Builder for Neglected Tropical Diseases (Coverage Survey Builder_v2.11.xls (live.com)) (CSB) was employed for the calculation of the sample size and the random selection of households in accordance with the recommendations of the WHO [6]. The requisite sample size was determined using the following formula:

$$n=\frac{\left(DEFF)({Z^{2}}_{1-\propto /2})(p)(1-p\right)}{\delta^{2}(1-r)}$$

- Estimated coverage of 69%;
- Precision of +/− 5%;
- Confidence interval of 95% or z-score of 1.96;
- Design effect of 4;
- Non-response rate of 10%;
- Average household size of 5.1 in rural areas [11].

The expected coverage is defined as the percentage of the population assumed to have ingested the medication. When the expected coverage is not known, the WHO suggests considering it as 50%. According to the WHO guidelines and to ensure that the sample size is sufficient to meet the study objectives, it is recommended to subtract at least 15 points from the percentage of the reported coverage. For this survey, an expected coverage of 69% was considered, given that the coverage reported by the program for 2022 was at least 84%. For the other sample size calculation parameters, the default values proposed by the WHO were used. The sample size generated by the CSB tool was estimated at 1460. A 10% increase was applied to account for the anticipated non-response, resulting in a minimum sample size of 1606 individuals.

### 2.5. Selection of Clusters, Households, and Respondents

In the Lukonga health zone, a comprehensive list of all villages was compiled, including the number of households and segments for each village. From this list, 30 villages were selected for inclusion in the study. Within each selected village, a segment was randomly selected for inclusion in the study. After listing all households within the selected segment, the households to be visited for the coverage survey were determined using a random list generated by the CSB. The survey included all residents of the selected households.

### 2.6. Data Collection and Analysis

The data collection process was conducted by independent investigators who were not involved in the MDA. All investigators received theoretical and practical training before the start of the survey. In each village, the survey team began by meeting with village leaders to explain the purpose of the study and obtain their consent to conduct the activity. Data collection was conducted using the mobile application Kobo Collect on Android phones, based on a standardized questionnaire. The study variables included socio-demographic characteristics (age, sex), ingestion of the drug combination (ivermectin + albendazole), occurrence and nature of the adverse events, and adherence to the MDA implementation guidelines (supervised intake). Data collection in each village was conducted by one investigator. Level of knowledge about MDA and lymphatic filariasis was measured. The latter was determined using a score constructed of six variables with equal weight in the score. These knowledge variables covered three main components: drug treatment, sanitation, and information about control of transmission and re-infection by self-protective behavior. A total score of 4–6 was considered high level of knowledge (good) and 0–3 as low level of knowledge (bad). The collected data were exported from the Kobo Collect server in Excel format, and data analysis was performed using Stata version 17 (Stata Corp, College Station, TX, USA).

### 2.7. Interpretation of Results

The observed coverage, or epidemiological coverage, is defined as the proportion of surveyed individuals who ingested the drug combination out of the total number of individuals surveyed. This value was compared to the WHO-recommended coverage threshold of 65% and the coverage reported by the Lukonga health zone [12].

Data analysis was conducted using Stata version 17 (Stata Corp, College Station, TX, USA). Descriptive analyses were used to report the demographic characteristics of participants. confidence intervals on proportions were calculated using the exact binomial method.

Prevalence by health area, education status, age groups, and gender was calculated, and the Clopper–Pearson binomial method was used to estimate 95% confidence intervals (CI). Prevalence estimates were adjusted for survey design using Stata command *svyset* (adjusting for probability of PSU selection within the country, probability of household selection within PSU, clustering at PSU and household, and finite population correction over all PSUs and households; standard errors were calculated using Taylor-linearized variance estimation).

In order to determine the household member traits that are linked to drug usage, a logistic regression model was used, with four specific characteristics being included as variables after checking for multicollinearity among the covariates, to obtain adjusted odds ratios (AORs) and their 95% confidence intervals (95% CIs). Variance inflation factors were calculated to test for multicollinearity, with the highest found to be 1.95. The significance level was set at *p* < 0.05.

### 2.8. Ethical Considerations

The survey was approved by the relevant authorities who considered the fundamental ethical principles of respect for persons, beneficence, and justice. This study was carried out in accordance with the principles outlined in the Helsinki Declaration. The protocol used in this study received ethical approval from the School of Health Ethics Committee (reference number: ESP/CE/74B/2023). Prior to the questionnaire’s administration, each household head was asked to provide verbal consent for the interview. The investigator also had to obtain verbal approval from the respondents at the beginning of each interview. All collected data were analyzed anonymously and confidentially. The principal investigator maintained the electronic and physical records in a secure location, with password-protected access for the computer and phones. 

## 3. Results

### 3.1. Characteristics of the Surveyed Individuals

The survey was conducted in 30 villages of the Lukonga health zone as planned. Overall, 1092 respondents out of 1132 participated in the survey (participation rate = 96.5%). A total of 329 households were visited, and 1092 individuals were surveyed. Approximately one-third of the participants were between 5 and 14 years old, and 54.8% of the participants were female. Nearly half of the respondents (47.6%) had completed primary education and 27.8% belonged to revival churches, while 31.2% identified with other religions. Regarding the relationship of the respondents with their household head, one-quarter of the total sample consisted of household heads, 19% were the wives of household heads, and nearly half were the children of household heads. In two-thirds of the surveyed households, or 64%, there were more than six individuals in residence, while in one-third of the households, or 36%, there were fewer than six individuals in residence.

The other characteristics are detailed in Table 1.

### 3.2. Therapeutic Coverage

A total of 1092 individuals were surveyed, and 1031 (94%) reported being present during the mass drug distribution in the community. Overall, 88% of the surveyed individuals reported receiving a visit from a community distributor (CD). Among the 1031 individuals present during the 2022 mass drug administration (MDA), 71% reported receiving treatment for lymphatic filariasis (ivermectin + albendazole), and 678 individuals reported ingesting the offered medications, resulting in an epidemiological coverage of 65.7% with a 95% confidence interval of 62.9–68.7, compared to the reported epidemiological coverage of 82.3%.

**Figure 2:**
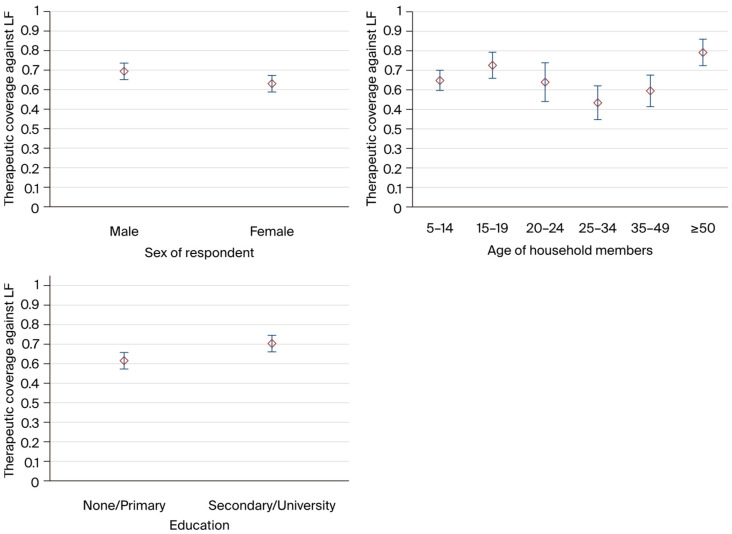
Therapeutic coverage for LF by respondent characteristics.

In terms of educational attainment, Figure 2 illustrates that the observed therapeutic coverage for those with a secondary/higher education level (70%) was higher than that observed for those with no/primary education level. The measured therapeutic coverage for the age group of 50 years and older was 78%, which was higher than the coverage for the other age groups (5 to 14 years, 15 to 19 years, 20 to 24 years, 25 to 34 years, and 35 to 49 years). It should be noted that the age group of 25 to 34 years had the lowest coverage, at less than 55%. Males had a higher measured therapeutic coverage (70%) than females (63%) (Figure 2). Religion did not affect the uptake of the medication.

**Figure 3:**
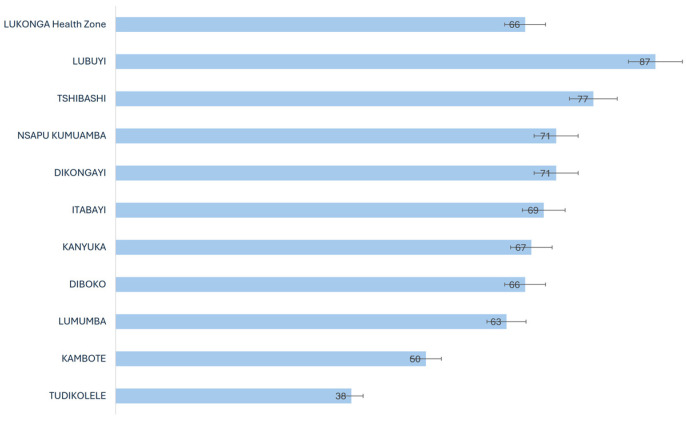
Distribution of therapeutic coverage by health areas.

Overall, 66% of the population present during the MDA reported having ingested ivermectin and albendazole for LF. The Lubuyi health area was the only area with an epidemiological coverage of over 80%. Seven health areas (Diboko, Dikongayi, Itabayi, Kanyuka, Kasasa, Nsapu, and Tshibashi) had an epidemiological coverage of 65% or more. Three health areas had lower epidemiological coverage (less than 65%), including one health area (Tudikolele) with a therapeutic coverage of 38% (Figure 3).

### 3.3. Reasons for Non-Compliance

**Figure 4:**
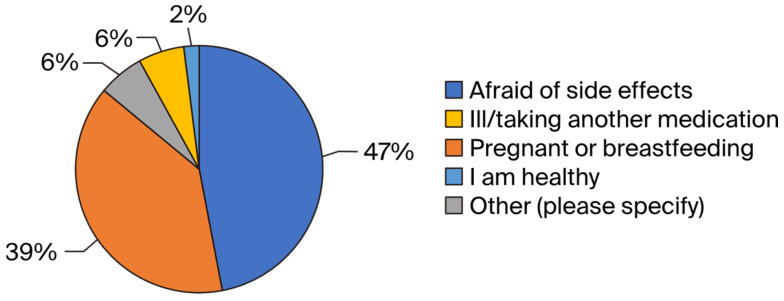
Distribution of respondents by reasons for non-consumption of medications.

The results of this study indicate that the primary reason for non-compliance among those who received the medications during the 2022 MDA was a fear of side effects related to ivermectin (47%), followed by being pregnant or breastfeeding (39%). Additional details are provided in Figure 4.

### 3.4. Adherence to Guidelines

With regard to the adherence to guidelines, it was observed that 66.4% of the 678 individuals who ingested the offered medications did so in the presence of the community distributors (CDs). This information is presented in Table 2.

### 3.5. Knowledge of LF and Adverse Effects

The most frequently cited symptom by the respondents was elephantiasis (78%), followed by hydrocele and other signs, each at 4.2%. It is noteworthy that 23% of the surveyed respondents who claimed to know about LF were not aware of its symptoms. With regard to the mode of transmission, only one individual (0.2%) was aware that the disease is transmitted by mosquito bites, while 0.8% of respondents were aware that the disease can be prevented through mass treatment. Among those who ingested the offered medications against LF, 14.8% reported experiencing side effects after receiving the treatment. The most frequently reported side effects were diarrhea (27%), headaches (19%), skin rashes (16%), stomach aches (13%), and other symptoms (31%) (Table 3).

### 3.6. Factors Associated with Uptake of MDA

**Figure 5:**
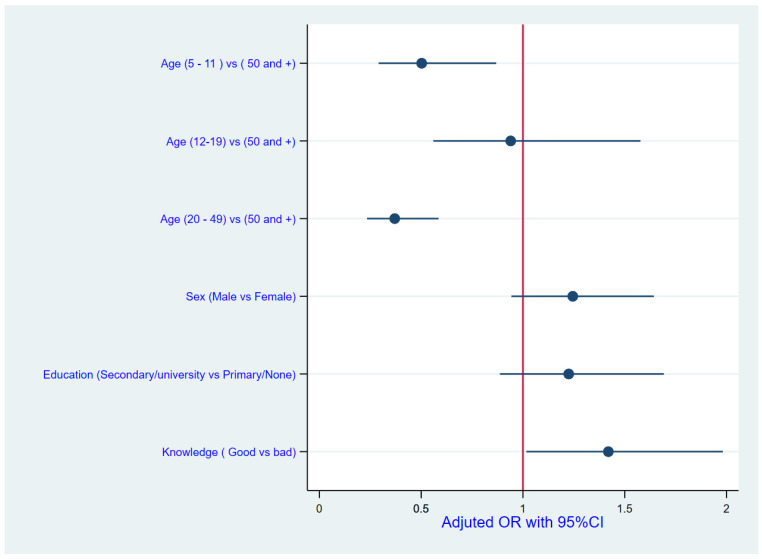
Diagram showing factors associated with uptake of MDA and their adjusted odds ratios (AORs).

In the multivariate analyses, the following characteristics were identified as factors associated with uptake of MDA: Age of household member and household member knowledge. With regard to knowledge, respondents who had good knowledge level were more likely to swallow drugs (OR 1.42, 95% CI 1.02–1.98). Older age group (age of 50 years or above) was associated with swallowing the drugs (Figure 5).

## 4. Discussion

Chemoprevention (PC) represents one of the principal intervention strategies used by programs designed to control and eradicate five neglected tropical diseases (NTDs), including lymphatic filariasis [6]. Monitoring PC coverage is typically based on the regularly reported coverage rates, which are calculated by aggregating the records from drug distributors and dividing by the estimated population requiring PC, according to census figures or reports from drug distributors, or, in some cases, the total population. While reported coverage is an essential tool for program monitoring, it is subject to errors resulting from incorrect estimates of the target population, weak health information systems, underreporting, and intentional inflation of the number of people treated.

In light of the observed bias in both EPI (older versions, pre-2015) and LQAS, the 2016 M&E Task Force recommended that national NTD programs implement coverage surveys using probabilistic sampling with segmentation (PSS). This method was also reviewed and approved by the Strategic and Technical Advisory Group for Neglected Tropical Diseases in 2016 for the evaluation of chemoprevention coverage [13]. The aforementioned methodology was used in the coverage survey conducted within the Lukonga health zone. The required sample size was not reached by the end of the survey. The observed epidemiological coverage in the Lukonga health zone may have been reached (as it falls in the confidence interval), but this is not certain, as the confidence interval extends to levels below those recommended by the WHO (≥65%) [13]. A study by Hoolageri [14] in the Bidar district yielded disparate results; of the 678 individuals who received the drug, 579 (85.4%) responded that they had ingested the drug. The observed therapeutic coverage in the survey, at 65.7% (95% CI: 62.9–68.7), appears to be lower than that reported by the Lukonga health zone. These findings are supported by the results from a post-treatment coverage survey for NTD-CTP conducted in six areas in the DRC by P. Akilimali [15]. The survey revealed an overall therapeutic coverage of 68%, with specific therapeutic coverages of 66.7% and 66.6% in the Bibanga and Kailo health zones, respectively.

The results of this survey indicate that the coverage achieved was close to the WHO target coverage threshold (less than 10 percentage points above the threshold), but the lower 95% one-sided confidence limit of the survey coverage was below the target coverage threshold. This suggests that the 2022 MDA cycle did not achieve the target threshold for effective coverage and that future improvements are necessary to increase the coverage [6,13].

Although the overall observed coverage slightly exceeded the WHO expected threshold, the analysis at the health area (AS) level revealed significant disparities. The Lubuyi health area was the only one with an epidemiological coverage of more than 80%. Seven health areas had epidemiological coverage of ≥65%, and three health areas presented lower epidemiological coverage (<65%), including one (Tudikolele health area) with an epidemiological coverage of 38%. The low coverage observed in certain villages may be attributed to inadequate geographical delineation of the areas to be treated [16]. This problem suggests the potential for inaccurate reporting of drug ingestion by distributors, resulting in inaccurate or outdated (overestimated) figures related to the total population and the population eligible for treatment. Alternatively, individuals from outside the area may receive medications from distributors and be recorded as residents of the area, necessitating intervention if resources permit [6]. Consequently, it is imperative that the health zone implement a monitoring system to assess therapeutic coverage at the level of each health area and document the reasons for any underperformance.

The reported coverage by the Lukonga health zone was not within the 95% confidence interval of the observed coverage after the survey, indicating that it is not validated by the survey results. This suggests a problem with the quality of the data reporting system. A more in-depth investigation into the reporting system is necessary to identify the sources of inaccuracy and propose corrective actions.

The results of this study indicate that approximately half of the individuals who received ivermectin and albendazole (47%) cited fear of ivermectin-related side effects as the reason for non-compliance with the treatment. Godale Lata’s study [17] yielded similar findings; in 120 households surveyed, 163 eligible individuals did not consume the medications, and the most frequently cited reason was a fear of side effects (45.38%). A study conducted by J. Jothula [18] revealed that 76.47% of the individuals who did not ingest the offered medications for LF cited a fear of side effects as the most common reason for non-compliance. The low level of community awareness before the MDA campaigns may be the root cause of non-compliance with the treatment. Therefore, there is a need to enhance awareness about adherence to the intake of distributed medications against LF in upcoming MDA campaigns to increase the coverage in the health zone.

This study demonstrates that adherence to the guidelines regarding directly observed treatment was not well respected. Indeed, out of a total of 678 individuals who ingested the offered medications, 66.4% reported doing so in front of the community distributors (CDs). These results are in contrast with the findings reported by several authors. In a study conducted in Burkina Faso, Kabre et al. reported that 99% of individuals who received the medications stated that they ingested them in front of the community distributors (CDs) [9]. Similarly, in a survey, P. Akilimali [15] reported that among those who consumed the medications, 95% stated that they did so in the presence of a distributor (report not yet published online).

It is recommended that direct observation of treatment be used regardless of the chosen administration mechanism, as it is the only method by which it can be ensured that individuals who receive the medications actually ingest them [19]. Ideally, the intake of medication should be supervised by the community distributors (CDs) during the mass drug administration (MDA). It is necessary to question the effectiveness of the training provided to CDs before the MDA and the supervision of these distributors during the campaign.

The results of this study indicate that only one individual (0.19%) among those who claimed to have knowledge of lymphatic filariasis (LF) was aware that the disease is transmitted by mosquito bites and were more likely to swallow the drug. Furthermore, only four individuals (0.84%) were aware that LF can be prevented through mass treatment. A study conducted by Professor P. Akilimali [15] yielded different results. Among those who claimed to know about LF, 23.6% knew that LF is transmitted by mosquito bites, and 47.4% knew that LF prevention is achieved through MDA [14]. It is true that cases of lymphatic filariasis are rare in the community, which may explain the low level of knowledge about LF among the respondents. These findings could be utilized by stakeholders to enhance the efficacy of messages disseminated during awareness campaigns on lymphatic filariasis.

Among those who ingested the medications offered as treatment against LF, only 14.8% reported experiencing side effects. Diarrhea accounted for 27% of the reported side effects. These results differ from those presented in several studies. A study conducted in Bengal reported that out of 467 subjects who had consumed the drug, only 30 (6.4%) experienced a side effect. The most frequently reported side effects were dizziness (43.3%), followed by nausea, vomiting, and headaches (26.7%, 23.3%, and 6.7%, respectively) [20]. A similar study conducted in Burkina Faso in two health districts revealed that a total of 98 individuals (0.3%) who ingested the medications reported developing side effects. Of these, 26 were in Fada N’Gourma and 72 were in Tenkodogo. The most frequently cited side effects by the respondents were breathing difficulties, drowsiness, diarrhea, and nausea [9]. Although these side effects are reported by respondents who ingested the medications offered (albendazole and ivermectin) against LF, it is important to note that these effects could be related to other issues besides the ingestion of the drugs. Therefore, community awareness before the upcoming MDA campaigns on the side effects related to the medications offered against LF during the MDA and their management is necessary.

However, this study did not assess individual adherence to mass treatment over several campaigns. Furthermore, the lack of financial resources prevented reaching the required sample size and obtaining the necessary qualitative information. The sample exhibits a lack of adequate representation of males. A significant number of males were not present during the campaign. The stated rationale was their relocation from the area, primarily to seek employment opportunities beyond the local community. The lack of equal representation in this survey may impact its coverage, as the gender analysis indicates that a greater proportion of men reported swallowing drugs compared to women. Additionally, if we incorporate the 31 individuals who were not present during the campaign into the denominator, the coverage will fall below the recommended 65%. To validate the coverage stated by the Lukonga health zone office and facilitate comparison, we have only included persons who were present in the village during the MDA.

## 5. Conclusions

The results of this survey indicate that coverage was close to the WHO target coverage threshold (less than 10 percentage points above the threshold). However, the coverage reported by the Lukonga health zone fell outside of the 95% confidence interval of the observed coverage after the survey, indicating that it is not validated by the survey results. Almost half of the individuals who received ivermectin and albendazole cited a fear of side effects related to ivermectin as the reason for non-compliance with the treatment. Only one individual among those who claimed to know about lymphatic filariasis was aware that the disease is transmitted by mosquito bites, and only four individuals, or 0.84%, were aware that the disease can be prevented by mass treatment. Among those who ingested the offered medications for LF, 14.8% reported experiencing side effects after taking the treatment, with diarrhea representing 27% of the reported side effects.

The key challenges to further expanding therapeutic coverage include the assessment of data quality, educating the population about the purpose and side effects, skills improvement, motivation of drug distributors, improvement of data reporting tools, accurate recording by community distributors (CDs) and reporting of non-residents who ingest the medications during the MDA, and harmonization of the numerator for calculating drug coverage in the health zone. Adherence to guidelines based on supervised medication intake, combined with individual treatment compliance over several years, could potentially interrupt LF transmission in the coming years.

Future research should be conducted to determine the population’s perception of MDA, evaluate adherence to MDA campaigns, and assess the quality of data transmitted in health areas with low coverage.

## Figures and Tables

**Table 1:** Sociodemographic characteristics of surveyed individuals during the MDA evaluation in the Lukonga health zone, conducted in December 2022.

Characteristics	Frequency	%
Age (years)		
5–14	358	32.8
≥15	734	67.2
Sex		
Female	598	54.8
Male	494	45.2
Education Level		
Never schooled	63	5.8
No response	1	0.1
Primary	520	47.6
Secondary	479	43.9
Higher	29	2.7
Household size	399	36.5
<6	693	63.5
≥6		
Religion of Respondent/Member		
Other	341	31.2
Catholic	218	20
Kimbanguist	83	7.6
Muslim	12	1.1
Protestant	124	11.4
Revivalist	304	27.8
No religion	10	0.9
Relationship to Household Head		
Household head	233	21.3
Spouse of household head	204	18.7
Son or daughter of household head	535	49.0
Grandchild of household head	64	5.9
Nephew or niece of household head	13	1.2
Brother or sister of household head	12	1.1
Parent of household head	12	1.1
Brother-in-law or sister-in-law of household head	10	0.9
Son-in-law or daughter-in-law of household head	8	0.7
Other	1	0.1
Respondent Status/Women *		
Neither pregnant nor breastfeeding	204	65.2
Pregnant	65	20.8
Breastfeeding	42	13.4
Do not know	2	0.6
Total	1092	100

* The percentage was calculated based on 313 female respondents aged 15 to 49 years.

**Table 2:** Coverage of ivermectin and albendazole treatment for lymphatic filariasis in the Lukonga health zone, 2022.

Characteristics	Frequency	%
Present during distribution		
Absent	60	5.5
Present	1031	94.4
Do not know	1	0.1
Total	1092	100
Received a visit from a CD		
No	129	12.5
Yes	902	87.5
Total	1031	100
Received treatment (ivermectin and albendazole)		
No	304	29.5
Yes	727	70.5
Total	1031	100
Ingested the offered medications (ivermectin and albendazole)		
No	353	34.2
Yes	678	65.7
Total	1031	100
Ingested the offered medications (ivermectin and albendazole) in front of the CD		
No	228	33.6
Yes	450	66.4
Total	678	100

**Table 3:** Knowledge of LF and reporting of adverse effects.

Characteristics	Frequency	%
Has heard about LF	478	43.8
Reported symptoms of the disease		
Elephantiasis	373	78.0
Hydrocele	20	4.2
Do not know	110	23.0
Others	20	4.2
Cannot remember	1	0.2
Total		110
LF transmission		
Knows that the disease is transmitted by mosquito bites	1	0.19
LF prevention		
Knows that the disease can be prevented by mass treatment	4	0.84
Good knowledge	557	51.0
Adverse effects		
Reported having adverse effects after taking treatment	100	14.8
Reported adverse effects		
Diarrhea	27	27.0
Headaches	19	19.0
Stomach aches	13	13.0
Skin rashes	16	16.0
Others	31	31.0

## Data Availability

The dataset used for analysis can be obtained upon reasonable request by writing an email to the corresponding author.
